# The Role of Endothelial Cells and TNF-Receptor Superfamily Members in Lymphoid Organogenesis and Function During Health and Inflammation

**DOI:** 10.3389/fimmu.2019.02700

**Published:** 2019-11-20

**Authors:** Kim C. M. Jeucken, Jasper J. Koning, Reina E. Mebius, Sander W. Tas

**Affiliations:** ^1^Amsterdam Rheumatology and Immunology Center (ARC), Department of Rheumatology and Clinical Immunology, Amsterdam UMC, University of Amsterdam, Amsterdam, Netherlands; ^2^Experimental Immunology, Amsterdam Infection and Immunity Institute, Amsterdam UMC, University of Amsterdam, Amsterdam, Netherlands; ^3^Department of Molecular Cell Biology and Immunology, Amsterdam Infection and Immunity Institute, Amsterdam UMC, Vrije Universiteit Amsterdam, Amsterdam, Netherlands

**Keywords:** LN development, TLS, inflammation, LN vasculature, endothelial cell, TNFR superfamily, NF-κB signaling

## Abstract

Lymph nodes (LNs) are crucial for the orchestration of immune responses. LN reactions depend on interactions between incoming and local immune cells, and stromal cells. To mediate these cellular interactions an organized vascular network within the LN exists. In general, the LN vasculature can be divided into two components: blood vessels, which include the specialized high endothelial venules that recruit lymphocytes from the bloodstream, and lymphatic vessels. Signaling via TNF receptor (R) superfamily (SF) members has been implicated as crucial for the development and function of LNs and the LN vasculature. In recent years the role of cell-specific signaling of TNFRSF members in different endothelial cell (EC) subsets and their roles in development and maintenance of lymphoid organs has been elucidated. Here, we discuss recent insights into EC-specific TNFRSF member signaling and highlight its importance in different EC subsets in LN organogenesis and function during health, and in lymphocyte activation and tertiary lymphoid structure formation during inflammation.

## Introduction

Lymph nodes (LNs) are positioned at strategic sites throughout the body where they are essential for initiating and shaping immune responses. Via the vascular system soluble factors and cells are transported from peripheral tissues into the LNs, which is crucial for the initiation of (adaptive) immune responses (1, 2). Lymph node reactions are tightly regulated and depend on interactions between incoming immune cells, local immune cells and LN stromal cells (3, 4).

**Figure 1:**
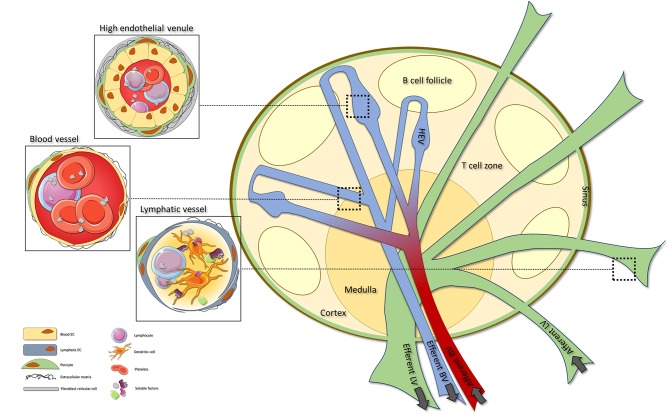
The lymph node vascular structure. Organization of the LN vasculature **(right)**. The LN vasculature consists of BVs, HEVs and LVs. BVs can be found throughout the whole LN, with specialized HEVs located within the T cell areas. Afferent LVs enter the LN where they transit into sinuses that ultimately exit via the efferent LV. HEVs are characterized by cuboidal ECs, pericyte coverage and a network of FRCs. HEV are specialized BVs that orchestrate extravasation of lymphocytes into the LN **(top left)**. In contrast to HEVs, normal BVs are lined by flat ECs and have minimal pericyte coverage **(middle left)**. LVs are characterized by overlapping lymphatic ECs that allow influx of (dendritic) cells and soluble factors into the vessel. Within the LN the LVs transit into sinuses that allow exit of the lymph fluid and its cells and soluble factors into the LN **(lower left)**. LN, lymph node; BV, blood vessel; HEV, high endothelial venule; LV, lymphatic vessels; FRC, fibroblastic reticular cell.

Generally, LNs can be divided into three areas; the cortex containing B cell follicles, the paracortex consisting predominantly of T cells, and the medulla representing the primary maturation site of antibody producing plasmablasts (1, 2). This spatial organization is orchestrated by LN stromal cells, that include distinct fibroblastic reticular cell subsets (FRCs) and endothelial cells (EC) (4, 5). The FRCs generate the highly organized scaffold network and comprises distinct stromal subsets such as T cell zone reticular cells (TRCs), marginal reticular cells (MRCs), follicular dendritic cells (FDCs), medullary reticular cells (MedRCs) and perivascular reticular cells (PRCs), reviewed elsewhere (5). Endothelial stromal cells can be divided into blood endothelial cells (BECs) and lymphatic endothelial cells (LECs). Importantly, the LN blood vessels include two main components: regular BVs and specialized high endothelial venules (HEVs) (Figure 1).

Arterial blood that enters the LN flows into capillaries which proceed into the network of HEVs. High endothelial venules located within the T cell areas are the sites were naïve lymphocytes leave the bloodstream to enter the LN and interact with local cells. After passing through the HEVs venous blood exits the LN via the efferent blood vessels (4, 6). Afferent lymphatic vessels (LVs) enter the LN and proceed into subcapsular sinuses (SCSs) that ultimately exit via the medullary LVs and efferent vessels (3, 7). Via the LVs, lymph fluid containing both soluble factors and cells is distributed by the SCSs to mediate interaction with local immune cells or stromal cells (3). Over the past years increasing attention has been paid to the role of endothelial cells (ECs) in LN development and function. More specifically, knowledge is being gained on EC-specific pathways necessary for LN organogenesis and function.

Among the signaling cascades that have been recognized as essential for LN development is signaling via the tumor necrosis factor receptor (TNFR) superfamily (SF). It is well-established that signaling of TNFRSF members in many cell types is required for proper LN development and function, including LN EC subsets.

Here, we present an overview of the EC-specific TNFRSF member signaling cascades that are important for LN organogenesis and development and maintenance of the different LN EC subsets. In addition, the importance of these cells and signaling pathways during inflammation are discussed, focusing on LN inflammatory reactions and development of tertiary lymphoid structures (TLSs). Lastly, we will discuss whether targeting of EC-specific TNFRSF member signaling may hold potential as a therapeutic target in the treatment of inflammatory diseases.

## Development of Lymph Nodes

Development of LNs starts around embryonic day (E) 9 at the same time that ECs start budding from the anterior cardinal vein and begin to express the lymphatic EC (LEC) marker lymphatic vessel endothelial hyaluronan receptor 1 (LYVE-1). Between E12.5-14.5, CD45^+^CD4^+^${\text{CD3}}^{-}{\text{α}}_{4}{\text{β}}_{7}^{+}$RORγt^+^IL-7Rα^+^ lymphoid tissue inducer (LTi) cells are recruited into the LN anlagen (8, 9) a process that is dependent on interactions with local stromal cells (10). Recently, it was demonstrated that CD4^−^ pre-LTi cells egress from venous vessels at locations where there is low coverage of smooth muscle cells (SMCs) (11). These pre-LTi cells then mature locally into CD4^+^ LTi cells that are transported to and retained at the site of LN development by the LVs (11).

Only once enough LTi cells appear and are retained at the site of the LN anlagen, definitive formation of LNs is started (12). Clustering of LTi cells that express both receptor activator of nuclear factor kappa B (RANK, also known as TNF-related activation-induced cytokine (TRANCE) receptor, and TNFRSF11A) and RANK ligand (RANKL, TRANCE, TNFSF11) leads to autocrine production of lymphotoxin α_1_β_2_ (LTα_1_β_2_, TNF-C) on LTi cells that engages the LTβ receptor (LTβR, TNFRSF3) on the surrounding stromal cells (13). Signaling via the RANKL-RANK and LTα_1_β_2_-LTβR axes creates a positive feedback loop, which leads to the recruitment of more LTi cells and expression of adhesion molecules by stromal cells. The differentiation of these mesenchymal lymphoid tissue organizer (LTo) cells is necessary to further support retention of LTi cells at the LN anlage (8, 14, 15). Next, the stromal LTo cells start to produce chemokines and cytokines that are necessary for definitive formation of LNs (8, 9, 15–18). Key molecules expressed by the LTo cells via the LTα_1_β_2_-LTβR signaling axis at this point include mucosal addressin cell adhesion molecule (MAdCAM)-1, vascular cell adhesion molecule (VCAM)-1, and intercellular adhesion molecule (ICAM)-1, and the chemokines C-X-C motif ligand (CXCL)13, C-C motif ligand (CCL) 19 and CCL21 (14, 19, 20). As the interactions between incoming (LTi) cells and local stromal (LTo) cells continue, a premature LN is formed that will ultimately develop into a fully functional LN a process that largely relies on TNFRSF member signaling.

### TNFR Superfamily Signaling in LN Development

**Figure 2:**
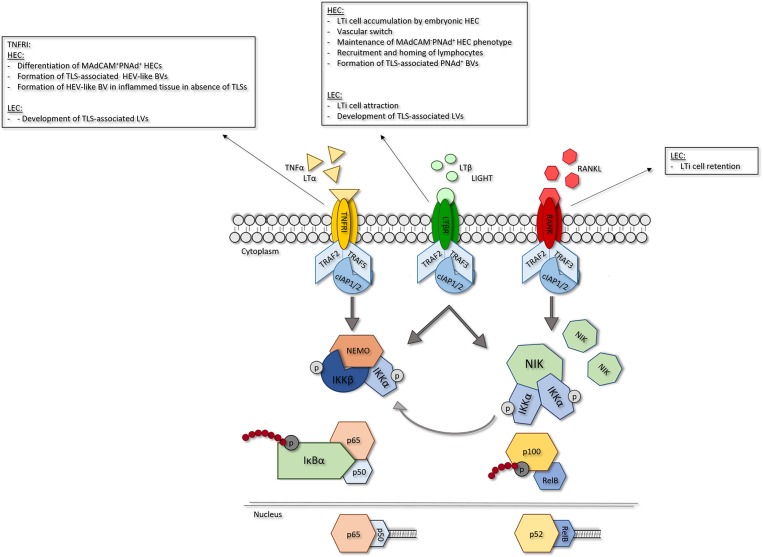
Role of EC-specific TNFRSF member signaling in LN development. Signaling via TNFRSF members leads to NF-κB signaling. In response to receptor ligation TRAF2 and−5 are degraded via activity of ciAPl/2 leading to activation of NF-κB signaling. Canonical NF-κB signaling is dependent on activity of the IKK complex. Activation of IKKleads to phosphorylation-induced degradation of Iκ*βα*, allowing nuclear translocation of p65/p50 dimers and transcription of canonical NF-κB target genes. Non-canonical NF-κB signaling is dependent on accumulation of NIK which forms a complex with IKKα. Activity of the NIK/IKKα complex leads to phosphorylation-induced degradation of plOO into p52 and p52/ReiB dimer translocation to the nucleus resulting in transcription of non-canonical NF-κB target genes. In EC, NIK/IKKα complex activity can also induce canonical NF-κB signaling. For each TNFR its role in the different LN EC subsets is shown. HEC, high endothelial cell; LEC, lymphatic endothelial cell; TNFRI, tumor necrosis factor receptor I; LTβR, lymphotoxin beta receptor; RANK, receptor activator of nuclear factor kappa B; LIGHT, TNFSF 14; TRAF, TNFR associated factor; clap, cellular inhibitor of apoptosis protein; NEMO, NF-κB essential modulator; IKK, IKB kinase; NIK; NF-κB inducing kinase.

Recently, more insights have been obtained into the importance of TNFRSF member signaling in ECs in the context of LN development. Ligation of TNFRSF members, including TNFRI, LTβR and RANK, predominantly activates nuclear factor kappa B (NF-κB) signaling (Figure 2). This signaling cascade can be divided into two distinct routes, the canonical and noncanonical NF-κB pathways (21). Canonical NF-κB signaling is dependent on activity of the IκB kinase (IKK) complex which is comprised of three subunits; IKKα, IKKβ and the regulatory subunit NF-κB essential modulator (NEMO, IKKγ). Activation of this complex leads to phosphorylation-induced degradation of IκBα resulting in the rapid nuclear translocation of the dimers p65(RelA)/p50 (22). Non-canonical NF-κB signaling is dependent on NF-κB inducing kinase (NIK) activity. Under homeostatic conditions, TNFR associated factor (TRAF) 2 and 3 (for LTβR and RANK signaling) or (for TNFRI signaling) 5 act as negative regulators of NIK by mediating its ubiquitination. Upon receptor ligation TRAF2 and TRAF3/5 are degraded by cellular inhibitor of apoptosis protein 1 and−2 (cIAP1/2), resulting in stabilization and accumulation of NIK, which enables its complex formation with IKKα (23). Activity of the NIK-IKKα complex leads to phosphorylation and degradation of p100 into p52 which can form dimers with RelB that are able to translocate to the nucleus. Although canonical and noncanonical NF-κB signaling are thought to follow two distinct routes, it has been shown that crosstalk exists between the two pathways. Recently, it was demonstrated that NIK can also drive canonical NF-κB signaling in EC (21), indicating that the effects of NIK are not solely restricted to the noncanonical pathway.

The importance of TNFRSF signaling in LN development and function is underlined by many studies in animal models with altered function of different components of the TNFR-NF-κB signaling cascades (Table 1). It has for instance been demonstrated that LTβR signaling is essential for viability, expansion and differentiation of LTo cells, as well as for recruitment of LTi cells (14, 19, 46). In addition, treating pregnant mice with anti-RANKL antibodies blocks LN formation in the offspring (47) and *Rankl*^−/−^ (43) and *Rank*^−/−^ (48) mice lack all LNs (25). Although early studies into TNFRSF member signaling were largely facilitated by global knock out (KO) models, the importance of EC-specific signaling via TNFRSF members in LN formation and function has recently been demonstrated with the aid of cell-specific KO models. For example, EC-specific *Ltbr* or *Nik* ablation impairs LN formation and function (12, 44, 45) and LEC-specific RANK signaling is shown to be essential for interaction with LTi cells (12). Thus, EC-specific signaling via members of the TNFRSF is crucial for LN development and function.

**Table 1:** Overview of LN deficiencies in TNFRSF member or TNFRSF member ligand KO mice.

	**Transgene**	**Peripheral LN**	**Mesenteric LN**	**References**
Global KO				
Receptors				
	*Ltbr^−/−^*	Absent	Absent	(24)
	*Rank^−/−^*	Absent	Absent	(25)
	*aly/aly*	Absent	Absent	(26, 27)
	*Nik^−/−^*	Absent	Absent	(28)
	*Ikka^−/−^*	Smaller	Smaller	(29–31)
	*Relb^−/−^*	Present, mild lymphoid depletion	NR^*^	(32, 33)
	*Nfkb2^−/−^*	Reduced iln	Present	(34, 35)
	*Nfkb1^−/−^*	Reduced iln	Present	(35)
	*Nfkb1^−/−^Nfkb2^−/−^*	Absent	Absent	(35)
	*Rela^−/−^Tnfr^−/−^*	Absent	Absent	(30)
	*Tnfr1^−/−^*	Similar to WT	Similar to WT	(29–31)
Ligands				
	*Ltb^−/−^*	Absent	Present	(29, 36–38)
	*Lta^−/−^*	Absent	Largerly absent	(36, 38–42)
	*Light^−/−^*	Present	NR^*^	(37)
	*Ltb^−/−^Light^−/−^*	Absent	Reduced compared to *Ltb-/-* mice	(37)
	*Rankl^−/−^*	Absent	Absent	(43)
	*Tnfa^−/−^*	Present	Present	(31, 41)
EC-specific KO				
	*Cdh5^*cre*^Nik^*fl*/*fl*^*	Reduced, smaller	NR^*^	(12)
	*Cdh5^*cre*^Ltbr^*fl*/*fl*^*	Reduced, smaller	Present	(12, 44, 45)
	*Cdh5^*cre*^Ltbr^*fl*/*fl*^Nik^*fl*/*fl*^*	Almost absent	NR^*^	(12)
	*Tek^*cre*^Ltbr^*fl*/*fl*^*	Smaller	Smaller	(45)
	*Tek^*cre*^Lyve1^*cre*^Ltbr^*fl*/*fl*^*	Smaller	Smaller	(45)
	*Lyve1^*cre*^Nik^*fl*/*fl*^*	Present	NR^*^	(12)
	*Lyve1^*cre*^Ltbr^*fl*/*fl*^*	Present	similar to WT	(12, 45)
	*Lyve1^*cre*^Ltbr^*fl*/*fl*^Nik^*fl*/*fl*^*	Reduced	NR^*^	(12)
	*Lyve1^*cre*^Rank^*fl*/*fl*^*	Reduced	NR^*^	(12)
	*Prox1^*cre*^Rank^*fl*/*fl*^*	*NR*	NR^*^	(12)

* *NR, not reported*.

### TNFR Superfamily Members in High Endothelial Venule Development and Function

Recruitment of lymphocytes from the blood into the LNs occurs via a network of post-capillary venules, the HEVs (6, 49). These specialized vessels that are largely restricted to lymphoid organs differ from regular BVs in that they are lined by cuboidal ECs, that express molecules critical for recruitment of immune cells (6, 50). Many of these key HEV markers are controlled via NF-κB signaling, including peripheral node addressin (PNAd), MadCAM-1, and other adhesion molecules (i.e., VCAM-1 and ICAM-1) and chemokines (i.e., CCL19 and CCL21) (51). In PLN, the most defining HEV marker is the adhesion molecule PNAd, which is a ligand for L-selectin^+^ lymphocytes. PNAd mediates rolling and tethering of lymphocytes on HEVs thereby allowing interaction of CCR7, expressed by lymphocytes, with CCL21 on HEVs (52). PNAd can be detected by binding of the MECA-79 antibody that binds to 6-sulpho sialyl Lewisx on extended core-1 branched O-linked sugars on CD34, glycosylation-dependent adhesion molecule (GlyCAM)-1, podocalyxin, endomucin and nepmucin (53, 54). Expression of PNAd is considered to be an exclusive feature of HEVs, whereas other markers expressed by HEV, such as MAdCAM-1 and CCL21, can also be expressed by other stromal cells, including LECs (55). Interestingly, it has recently been shown that expression of these markers by HEV can be relatively heterogeneous during LN homeostasis (56).

True HEVs are not present until birth when EC begin to form a network in the T cell areas surrounding the B cell follicles, which is critical for completion of the LN infrastructure and function (12). Initiation of HEV development occurs by ligation of the TNFRs on blood ECs (BECs) leading to vasculature growth and HEV formation, followed by entry of lymphocytes into the LN (44, 57, 58) and completion of the HEV network around postnatal day (P) 4 (19). It has been proposed that initial canonical NF-κB signaling via LTα (TNF-β)-TNFRI interaction generates MadCAM-1^+^PNAd^+^ flat HEVs, and that sustained LTβR and downstream non-canonical NF-κB signaling induces development of MadCAM-1^−^PNAd^+^ cuboidal HECs (6). A key event in the maturation of HEVs is the simultaneous upregulation of PNAd and downregulation of MadCAM-1 a process known as the vascular addressin switch (9, 50, 59). Until time of birth HEVs express both MAdCAM-1 and PNAd (59). Next, over a period of 4 weeks there is a change in addressing expression, eventually leading to MAdCAM-1^−^PNAd^+^ HEV (59). It is suggested that this switch is induced by LTα_1_β_2_ expressing DCs originating from the gut (60, 61). These DCs engage the LTβR on immature HEVs resulting in a decrease of MAdCAM-1 expression and an increase in PNAd expression and subsequent homing of lymphocytes (60, 61). Of note, while MAdCAM-1 expression is completely downregulated in PLN HEVs, HEVs in LN associated with mucosal tissues continue to express MAdCAM-1 alongside PNAd (6, 51). Once the switch from MadCAM-1^+^PNAd^−^ to MadCAM-1^−^PNAd^+^ HEVs is completed, the LTβR needs to be frequently, if not constantly, engaged in order to maintain a mature HEV phenotype (44, 57, 62). It has been shown that CD11c^+^ DC can fulfill this function as they make frequent contact with the HEVs to establish the LTα_1_β_2_-LTβR interaction and downstream NF-κB signaling that is essential to control PNAd and MAdCAM-1 levels (57, 60, 63). It is thought that LTβR signaling is the dominant receptor in maintaining the HEV phenotype since LTβR, but not TNFRI, blockade leads to the loss of several HEV-specific markers (57). For instance, interfering with LTβR signaling during PLN homeostasis influences expression of several genes involved in cell adhesion and expression of HEV markers such as Glycam-1 while having no effect on others, including CCL21 (56).

Although true HEVs only start to develop after birth, a recent study identified a small subset of EC with characteristics of HECs already present during embryonic development (45). These ECs express genes, including *Madcam-1, Cxcl13*, and *Ccl21*, which are also expressed by HECs present after birth (45). Targeting these embryonic HECs in a *Tek*^*cre*^*Ltbr*^*fl*/*fl*^ model resulted in a reduction of LTi cell accumulation and subsequent defects in LN maturation suggesting that LTβR signaling in embryonic HECs may play a role in LN formation during embryogenesis (45).

### TNFR Superfamily Members in Lymphatic Vasculature Development and Function

Lymphatic vessels are blind ending, thin walled, vessels that are the first entry points for antigen and antigen presenting cells (APC) from tissues into the LNs (64). Characteristic LV markers include LYVE-1, prospero homeobox protein 1 (PROX-1), podoplanin (PDPN), CCL21 and vascular endothelial growth factor (VEGF) receptors (R)−2 and−3 (55, 65). Via extensions into the T and B cell areas LVs are able to centralize antigen presentation, as well as lymphocyte distribution and migration within the LN, either by simply delivering soluble factors or cells, or by acting as APCs themselves (1, 66–69).

Afferent LVs originating from the peripheral tissue branch into the SCSs located directly underneath the LN capsule, extend into the T and B cell areas, and exit as efferent vessels (7, 70). Via these extensions LVs are able to centralize antigen presentation, as well as lymphocyte distribution and migration within the LN, either by simply delivering soluble factors or cells, or by acting as APCs themselves (1, 66–69).

Unlike formation of HEVs, LV formation is already initiated within the same timeframe as LN formation (8, 9, 11). Details for LV formation have mostly been studied in inguinal (i) LN as these can already be found prenatally. In iLN the first event in the development of LVs is the formation of a capillary-like plexus (11, 71) which matures into LYVE-1^low^VEGFR^+^ collecting LVs between E15.5-E16.5 (11, 72) ultimately forming a lymphatic cup that surrounds the developing LN anlagen by E20.5 (11). Remodeling of initial LVs is dependent on engagement of VEGFR-3 on LECs by VEGF-C produced by surrounding stromal cells in a LTβR-dependent manner (20, 73). While the mechanisms underlying VEGFR-3 expression by LECs are not fully understood, at least one study identified VEGFR-3 as downstream target of canonical NF-κB signaling (74).

Recently, the details of the sequence of events and the importance of LECs during iLN development have become clear (11). Although starting within the same timeframe, initial formation of the LN anlagen is independent of LEC differentiation (11, 75). Differentiation of LECs into collecting LVs is important for uptake and transport of mature CD4^+^ LTi cells into the iLN anlagen. In addition, iLN size is also defined by the number of cells that can be retained a process that depends on CXCR5-CXCL13 mediated interaction between LT${\text{α}}_{1}{\text{β}}_{2}^{+}$ LTi cells and LTβR expressing LTo cells (10, 11). CXCL13 expression by LTo cells is known to be indispensable for LTi cell retention and it is now clear that LTβR signaling together with interstitial fluid flow regulated by collecting LVs can induce LTo cell CXCL13 expression (11).

Recently, the functions of LECs in LN development have become more clear, aided by studies focusing on the role of LEC-specific TNFRSF member signaling (12, 45, 76). It was shown that more than half of *LYVE-1*^*Cre*^*Ltbr*^*fl*/*fl*^*Nik*^*fl*/*fl*^ mice have a loss of PLNs due to incapacity to attract sufficient LTi cells to expand the LN anlagen (12). Interestingly, single deletion of either *Ltbr* or *Nik* in LECs does not affect the number of PLN formed (12, 45), indicating that compensatory mechanisms may take over when either LTβR or NIK is not functional. In addition, it was shown that LEC-specific NIK deletion impairs the recruitment of B cells into the PLN and it is suggested that this might be due to reduced CXCL13 expression (77). Consequently, LTβR-NIK signaling in LEC may be crucial for the expansion and maturation of fully functional LNs. In addition to LTβR signaling, LEC-specific RANK signaling is involved in LN formation. It is suggested that interfering with RANK signaling reduces expression of ICAM-1 and VCAM-1 on LECs, leading to impaired LTi cell retention in the developing LN anlagen (12).

For a long time, the exact role for LECs in LN development was not completely clear. A recent study using *LYVE-1*^*cre*^*Rank*^*fl*/*fl*^ mice suggests that recruitment of LTi cells by LECs is the first step leading to formation of the LN anlagen (12, 78), whereas it has also been shown that formation of the LN anlagen can be initiated in absence of LECs (11, 75). The recent findings by Bovay et al. provide evidence that although LTi cells may form an initial LN anlage independently of LECs, LECs are crucial for the formation of definitive LNs by transporting CD4^+^ LTi cells that have egressed from venous locations to the LN anlagen (11).

## The Lymph Node Vasculature During Inflammation

### Blood Vessels

During inflammation soluble factors, antigens, DCs and/or lymphocytes are drained from the site of insult into the LNs (79). The inflammatory response that follows leads to an increase in LN size which mostly relies on remodeling of the blood vasculature (80–82) and expansion of the LV (83). Once inflammation is resolved, the vasculature and with that the complete LN, returns to its homeostatic condition (79).

Under inflammatory conditions the number and characteristics of the immune cells infiltrating the draining LN is altered, which triggers changes in the local vasculature. These changes include general expansion of the LN blood vasculature to increase blood supply and influx of lymphocytes. Expansion of the HEV network is regulated by DCs producing VEGF-A, which directly stimulates HEV growth, and LTα_1_β_2_ which ligates LTβR fibroblastic stromal cells leading to production of more VEGF-A (57, 81, 84). In addition to alterations in the volume of lymphocyte influx, the composition of the influx is also changed which is predominantly caused by a change in the expression of homing associated molecules and inflammatory chemokines, that enable recruitment of (activated) immune cells that are not recruited under homeostatic conditions (50, 85).

Several studies showed that phenotypic changes occur in HEVs during inflammation without compromising their ability to recruit naïve lymphocytes (56, 82, 86). These changes suggest a reversal toward a more immature state, where downregulation of mature HEVs genes coincides with temporary upregulation of more immature HEVs genes like MAdCAM-1 (56, 57, 62, 87). It is proposed that this change in gene expression aids the entry of LTi and other innate lymphoid cells (ILC) which are necessary for eventual restoration of the homeostatic architecture (87). More recently, using single-cell analysis, detailed insights into the temporary changes occurring in PLN HECs during inflammation were obtained. Generally, it was shown that temporary changes include downregulation of several mature HEV genes like *Glycam-1* and *Ccl21* during the first few days after immunization, with a restoration in expression by day 7 post immunization. However, expression of other HEV markers, including MECA-79 is maintained, most likely because these are essential for recruitment of naïve lymphocytes into PLNs. In addition, there is a temporal upregulation of other adhesion molecules, like MAdCAM-1 and E- and P-selectin (56).

This remodeling of HEVs and thereby the entire composition of the LN during inflammatory responses is largely dependent on engagement of LTβR mediated by LTα_1_β_2_-expressing B cells, among others, and to a lesser extent on classical growth factors like VEGF-A (57, 81, 88).

Importantly, during inflammation LTα_1_β_2_-LTβR mediated crosstalk between HEVs and LVs exists: HEVs are demonstrated to locate around LV, and in some cases HECs that express LYVE-1 are observed, indicating that during inflammation HEVs might adopt certain features of LVs (56, 62). These double positive vessels are mainly found during the first few days after immunization and eventually disappear. Interestingly, changes in the LV network seem to occur in parallel with changes in HEVs, since during the first days following immunization there is a temporary decrease in LV function, which is restored in parallel with HEV recovery (62).

### Lymphatic Vessels

Lymphatic ECs are among the first cells to react to inflammatory insults and expansion of the local LVs via lymphangiogenesis plays an important role during inflammation. Lymphangiogenesis enables increased transport of fluid, immune cells and APCs from the site of inflammation into the LN to initiate the first immune response (62, 89). Lymphatic vessel remodeling is not only apparent at the site of insult, but also in the draining LNs (90).

Interestingly, during inflammation there seems to be a strict spatial and temporal regulation of LN lymphangiogenesis that is thought to regulate the sequential regulation of DCs and T cells (90, 91); lymphangiogenesis is first observed in the SCSs, and only later in the cortical and medullary sinuses (91). In addition to morphological changes, LECs also alter gene expression during inflammatory responses including upregulation of many NF-κB related genes including *Icam-1, Vcam-1, Cxcl12* and *Ccl21* (55). Of these chemokines, CCL21 is best characterized: it is upregulated under inflammatory conditions and known to mediate migration of CCR7^+^ immune cells (92). Together, this suggests that LVs are important for initiation and shaping of immune responses within the LN. Similar to HEV remodeling, lymphangiogenesis can be reduced by interfering with LTβR signaling or by depleting (LTα_1_β_2_-expressing) B cells, suggesting concerted action of the LTβR and B cells in lymphangiogenesis and HEVs plasticity (62, 93).

Once inflammation is resolved there is a return to normal LN homeostasis, including reversal of the vasculature. However, if inflammation persists, the inflammatory phenotype of the LN is maintained, contributing to persistence of inflammation (94, 95).

## Tertiary Lymphoid Structure Formation

In addition to changes in LNs, persistent inflammation and antigen stimulation can lead to local formation of lymphoid like tissues, which can vary from unorganized cellular infiltrates to highly organized tertiary lymphoid structures (TLSs) (96). TLSs resemble LNs, in that they are characterized by a network of activated stromal cells and FDCs, as well as the presence of PNAd^+^ ECs that represent the HECs in LN. In addition, TLSs are composed of distinct B and T cell areas. But in contrast to LNs, TLSs lack a capsule and independent afferent LVs (96, 97). Emerging evidence suggests that, in addition to structural similarities, there is also overlap between the cells and molecules directing LN and TLS development. TLSs have ectopic expression of lymphoid chemokines and cytokines, including LTα, LTβ, CXCL13, CXCL12, CCL21, and CCL19 (98–100). The development and function of TLSs, as well as the cellular players involved have been extensively reviewed elsewhere (96, 97, 99–101) and therefore we will mainly focus on the role of TNFRSF members in TLSs and their associated vessels.

Many animal studies have shown that overexpression of a single inflammatory cytokine or homeostatic chemokine is sufficient to initiate TLS development and TNFRSF members and their ligands are among the main players [reviewed in (97)]. More than 20 years ago, the group of Ruddle showed that mice overexpressing LTα_1_β_2_ or TNFα under the rat insulin promotor (RIP-LTα_1_β_2_ and RIP-TNFα transgenic (tg) mice, respectively) spontaneously develop infiltrates consisting of T and B cells in the pancreas (102). Additional studies further investigated the role of TNFRSF members in TLS development and function. Overall, these studies demonstrated that signaling through TNFRSF members plays a critical role in the development of TLSs. Signaling through the LTα/TNF-TNFRI axis is sufficient to initiate TLS formation, but LTα_1_β_2_-LTβR signaling leads to larger and more organized TLS with higher expression of TLS associated cytokines and chemokines (i.e., CXCL13, CCL19, CCL21) (98, 102, 103). It is thought that LTα_1_β_2_ and other cytokines and chemokines produced by immune cells acting like LTi cells, ligate receptors on local stromal cells which are then stimulated to differentiate into a lymphoid stromal cell-like phenotype that can drive TLS formation (97, 104–106). So, interaction between immune cells acting like LTi cells and stromal cells acting like LTo is crucial for the development of TLSs. Together, this suggests that the signaling pathways and players involved in TLS and LN development largely overlap.

### Tertiary Lymphoid Structure Associated High Endothelial Venules

As already mentioned, one of the characteristics of TLSs is the presence of PNAd^+^ HEC-like cells. During chronic inflammation the blood vasculature undergoes remodeling in order to recruit immune cells into the inflamed tissue. This remodeling involves upregulation of adhesion molecules and production of chemoattractants by BECs (107–109). Ultimately, as inflammation persists, these BECs can differentiate into PNAd^+^ HEC-like cells that orchestrate extravasation of L-selectin^+^ and CCR7^+^ immune cells into the TLSs (97).

In general, TLS associated PNAd^+^ BVs have the same function as LN HEVs, namely recruitment of (naïve) immune cells into the tissue to mediate interaction with cognate antigens leading to immune cell activation and memory cell formation (98, 102, 110–112). Interestingly, in TLSs the consequences of these interactions may be different depending on the pathology. For example, in autoimmune diseases like rheumatoid arthritis, recruitment and local activation of autoreactive B and plasma cells is likely to exacerbate disease (113, 114). In contrast, in infections recruitment of immune cells to the site of infection might aid in its resolution by limiting spreading of the microorganisms and confining the immune reaction (6, 96). In the case of cancer, PNAd^+^ BVs may act anti-tumoral via recruitment of naïve and memory T cells (115–117), but they can also facilitate immune evasion by recruitment of immunosuppressive cells, like regulatory T cells (50).

Similar to LN HEVs, formation of PNAd^+^ BVs in TLSs seems to be LTβR dependent. It has been shown that initiation of TLS-PNAd^+^ BVs occurs via the LTα-TNFRI-canonical NF-κB axis leading to MAdCAM-1^+^ BVs and that LTα_1_β_2_-LTβR-noncanonical NF-κB signaling generates PNAd^+^ BVs (29, 98, 118). This is underlined by the finding that TLS PNAd^+^ BVs located in the synovial tissue of rheumatoid arthritis patients are NIK^+^ comparable to LN HEVs (119). In addition, in the synovial tissue of these patients ILC3s were found, and although present in very small numbers, they might act similar to LTi cells in the formation of PNAd^+^ BVs (119). Although LTβR signaling is typically required for the formation of mature HEVs, when PNAd^+^ BVs are formed in absence of real TLSs, PNAd^+^ BVs can also develop via LTα-TNFRI signaling, independent of LTβR signaling (120). Together, these data suggest that the same mechanisms that lead to formation of LN HEVs are involved in the formation of TLS associated PNAd^+^ BVs. However, the function of these vessels is shown to vary between pathologies, making it hard to predict the outcome of targeting HEVs during disease.

### Tertiary Lymphoid Structure Associated Lymphatic Vessels

The role of LVs in inflammation has long been recognized; i.e., LVs play an immunoregulatory role in inflammation via fluid drainage, scavenging of inflammatory chemokines and suppression of DC maturation (121, 122). During chronic inflammation, it is thought that the lymphangiogenic process is altered and that the amount of infiltrating immune cells exceeds the draining capacity of LVs. Lack of efficient draining might lead to local persistence of antigens and immune cells, and non-functional LVs might compete with functional LVs leading to trapping of immune cells at the site of insult, favoring development or maintenance of TLSs (123, 124).

Although LYVE-1^+^ Prox-1^+^, PDPN^+^CCL21^+^ vessels are found in TLSs of many pathologies, their exact role and function is not well-understood (125, 126). They often contain cells, implying that they have a role as transporters. On the other hand, these vessels can be so crowded with cells that their drainage and efferent functions are likely impaired (127). In contrast to LN LVs, it seems less probable that TLS LVs are necessary for antigen transport since TLSs are already characterized by local presence of antigens. The source of these antigens may be the LVs itself, since it has been shown that LECs are capable of antigen archiving and –presentation (69). It has also been proposed that within TLS, LECs might act as APCs to induce tolerance or T cell activation (125).

In mice lacking LTβR, TNFRI or LTα, the development of LVs in the inflamed area is impaired, while the surrounding LVs remain largely unaffected (128, 129). It was shown that LTα is sufficient to induce lymphangiogenesis even before the onset of organized TLSs, whereas LTα_1_β_2_ is not required (129). Of note, it was shown that LTα_1_β_2_ might even negatively regulate lymphangiogenesis since *Ltb*^−/−^ mice exhibit increased lymphangiogenesis, which may be due to the fact that mice lacking LTα_1_β_2_ have more LTα available to form trimers that bind to TNFRI (129). Together this indicates that TNFRSF member signaling is involved in inflammatory development of LVs, but not for the maintenance of the existing lymphatic vasculature.

The role of RANK signaling in TLS associated LVs has not yet been fully addressed, but with the recent evidence demonstrating an important role for that RANK signaling in LECs in the development of mature functional LNs (12), it will be of great interest to investigate the importance of RANK signaling in the formation and function of TLS.

## Concluding Remarks and Future Perspectives

It is clear that signaling through TNFRSF members is crucial for development and functioning of LNs and TLSs, both in health and disease. Here, we emphasized the importance of EC-specific TNFRSF member signaling cascades in these processes.

It is clear that TNFRSF signaling, mainly via the LTβR, in BECs is crucial for their differentiation into HECs, which subsequently regulate recruitment of lymphocytes into the LNs. In addition, the same TNFRSF-mediated mechanisms are involved in formation of TLS associated PNAd^+^ BVs.

Although considerably less is known about TNFRSF member signaling in LVs, recent data does point toward an important role for these pathways in LEC function in LN development and function. A prominent role seems to be reserved for signaling via RANK, although LTβR signaling is also likely to be involved. Interestingly, different mechanisms may be involved in functioning of LECs in LNs and TLSs.

The currently available studies aimed at unraveling the role of signaling pathways in LN EC subsets already prompt possible refinements of the existing models for LN development and the role of TNFRSF member signaling in ECs within this process, but there are still several outstanding questions. One of these questions is to what extent the timing of TNFRSF signaling is important, since it is not fully understood at what exact time point signaling events in specific EC subsets are crucial for the development of fully functional lymphoid organs. In this respect, the development of EC-specific conditional KO mice is very promising and aid tremendously in addressing this issue.

In line with this, it will also be interesting to see if interfering with specific signaling pathways in EC subsets holds therapeutic potential in treatment of chronic inflammatory diseases and other pathologies. Several clues point toward this, for example the finding that NIK can be detected in TLS associated PNAd^+^ BVs and other blood vessels in inflamed tissues, but not in healthy tissues (130). Here, conditional EC-specific KO models in combination with disease models will allow to dissect the importance of TNFRSF members and their ligands in EC both in LN and target tissues before onset and during active disease.

It is likely to assume that in the near future many outstanding questions will be answered. With the current possibilities in animal models and advanced techniques to compare patients with healthy individuals, it is only a matter of time until deeper insights into the cell specific signaling pathways in development and maintenance of LNs and TLSs are gained. More importantly, it will be of great interest to uncover how these pathways contribute to disease and whether they hold therapeutic potential.

## Author Contributions

KJ: conceived the general idea, wrote the manuscript, and created the figures. JK and RM: provided expert opinion/knowledge input, and co-wrote the manuscript. ST: conceived the general idea, provided expert opinion/knowledge input, and co-wrote the manuscript.

### Conflict of Interest

The authors declare that the research was conducted in the absence of any commercial or financial relationships that could be construed as a potential conflict of interest.
